# Recent Advances in Self-Powered Piezoelectric and Triboelectric Sensors: From Material and Structure Design to Frontier Applications of Artificial Intelligence

**DOI:** 10.3390/s21248422

**Published:** 2021-12-17

**Authors:** Zetian Yang, Zhongtai Zhu, Zixuan Chen, Mingjia Liu, Binbin Zhao, Yansong Liu, Zefei Cheng, Shuo Wang, Weidong Yang, Tao Yu

**Affiliations:** 1 School of Aerospace Engineering and Applied Mechanics, Tongji University, Shanghai 200092, China; 1930905@tongji.edu.cn (Z.Y.); 20666106@tongji.edu.cn (Z.Z.); zixuanchen@tongji.edu.cn (Z.C.); 2130895@tongji.edu.cn (M.L.); 2130897@tongji.edu.cn (B.Z.); 2130896@tongji.edu.cn (Y.L.); czfshr@163.com (Z.C.); wangshuo960228@163.com (S.W.); yutao@tongji.edu.cn (T.Y.); 2 The Shanghai Key Laboratory of Space Mapping and Remote Sensing for Planetary Exploration, Tongji University, Shanghai 200092, China

**Keywords:** self-power, piezoelectric, triboelectric, sensor, AI, flexible electronics

## Abstract

The development of artificial intelligence and the Internet of things has motivated extensive research on self-powered flexible sensors. The conventional sensor must be powered by a battery device, while innovative self-powered sensors can provide power for the sensing device. Self-powered flexible sensors can have higher mobility, wider distribution, and even wireless operation, while solving the problem of the limited life of the battery so that it can be continuously operated and widely utilized. In recent years, the studies on piezoelectric nanogenerators (PENGs) and triboelectric nanogenerators (TENGs) have mainly concentrated on self-powered flexible sensors. Self-powered flexible sensors based on PENGs and TENGs have been reported as sensing devices in many application fields, such as human health monitoring, environmental monitoring, wearable devices, electronic skin, human–machine interfaces, robots, and intelligent transportation and cities. This review summarizes the development process of the sensor in terms of material design and structural optimization, as well as introduces its frontier applications in related fields. We also look forward to the development prospects and future of self-powered flexible sensors.

## 1. Introduction

Multifunctional flexible sensors have been widely used in human health detection, intelligent robots, and other fields in recent years because of their characteristics of high sensitivity, high resolution, and low cost [1]. Scientists have achieved stretchable [2], self-healing [3], degradable [4], and multifunctional flexible sensors [5] through different kinds of material design. However, conventional flexible sensors used to require external energy devices to provide energy to obtain the signal of sensing. Energy harvesters [6,7] are an integral unit of the self-powered multifunctional sensor. In response to this challenge, scientists continue to study the use of energy in the natural world to convert energy into the sensor to fabricate the realization of self-powered sensors.

In the context of global sustainable development, energy-storage devices with high performance [8,9,10,11,12,13,14,15] have attracted widespread attention. With the development of multifunctional sensors, higher requirements have been put forward for their related performance [16,17,18,19,20,21] such as obtaining real-time and effective energy without any interruption. The proposal of PENGs [22] and TENGs [23] can effectively convert pressure and friction into energy output. Furthermore, no matter how small the deformation of the mechanical force is, it can be converted into adequate energy [24,25,26]. This dramatically promotes the scope of application. The emergence of TENGs and PENGs provides a stable and effective way to convert weak mechanical energy into electrical energy. PENG realizes the energy conversion based on the piezoelectric effect. The piezoelectric effect refers to the deformation of piezoelectric materials under the action of mechanical force, whereby the phenomenon of internal polarization occurs, and a charge with the opposite sign appears on the surface of the material. The charge density generated by the force is proportional to the magnitude of the force. In fact, the piezoelectric effect is a process in which the mechanical energy is converted into electrical energy. The unique feature of the piezoelectric effect is that it is reversible [21]. The basic principle of the TENG is based on the triboelectric effect. When the electrodes are in contact, the two films with very different electronegativity are rubbed; then, when they are separated, they carry opposite charges and form a potential difference. The potential difference causes electrons to flow between the two electrodes to balance the electrostatic potential difference between the films. Once the two contact surfaces overlap again, the potential difference generated by the triboelectric charge disappears, such that the electrons flow in the opposite direction. Through such constant contact and separation, the output end of the friction generator outputs alternating current pulse signals, thereby outputting electric energy to the outside [23]. These two kinds of nanogenerators have been widely studied because of their advantages [27], such as light weight, small size, wide range of materials, high output power, and stability. The output performance of TENGs and PENGs has been dramatically improved due to the progress in theory and practice [28]. Moreover, some new materials have been used to manufacture high-performance self-powered multifunctional sensors [29,30,31,32]. Therefore, the application of TENGs and PENGs as self-powered multifunctional sensors has been greatly developed [29,30,31,32,33,34]. They can not only be used as energy storage devices but also realize the function of sensing. These electrical signals can be connected to the computer to realize human–machine interfaces.

This paper summarizes self-powered flexible sensors based on TENGs and PENGs from two aspects: material and structure design. While summarizing their applications in electronic skin, human–machine interfaces, and robots, we also look forward to the direction of future development.

## 2. Material Design

### 2.1. Stretchability

Stretchability is one of the important advantages of flexible sensors [35]. Conventional self-powered sensors based on a hard and brittle substrate cannot be used in large mechanical deformations. However, a stretchable flexible sensor can solve this problem, being of more practical value, including wearable technology and E-skin of soft robots [36,37,38,39]. Furthermore, a flexible sensor with stretchability can maintain a better sensing function even when stretched to several times its natural length [40]. Current self-powered stretchable flexible sensors are mainly divided into two structures: single-fiber-shaped and flat-shaped sensors.

**Figure 1:**
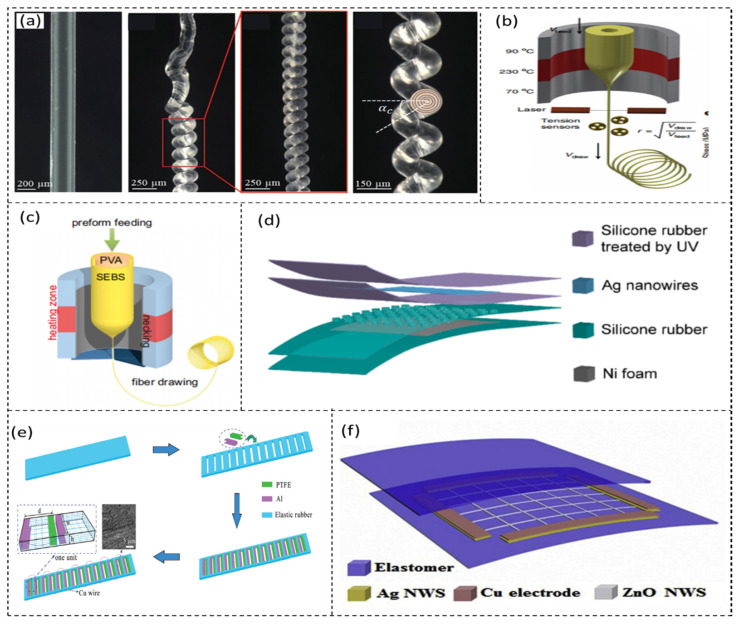
(**a**) Photos of PVDF melt-spun fiber. Reprinted from Ref. [41]. (**b**) Schematic of thermal drawing process. Reprinted from Ref. [42]. (**c**) Schematic of the fiber drawing process. Reprinted from Ref. [44]. (**d**) Schematic of DS-TENG. Reprinted with permission from Ref. [45]. Copyright 2019 American Chemical Society. (**e**) Schematic of SF-TENG. Reprinted with permission from Ref. [46]. Copyright 2018 John Wiley and Sons. (**f**) Schematic of polyionic triboelectric patch. Reprinted with permission from Ref. [48]. Copyright 2019 Elsevier.

(1) Fiber-shaped structure. The structure of a single fiber can achieve excellent stretchability. Highly sensitive and stretchable fibers can be woven into smart fabrics and wearable electronic products. Mokhtari et al. [41] fabricated nanostructured hybrid polyvinylidene fluoride (PVDF)/reduced graphene oxide (rGO)/barium–titanium oxide (BT) piezoelectric coiled fibers by melt spinning and knit and coil fabrication. The twisted fiber endured axial extension up to 100% strain. The peak voltage output reached 1.3 V under the strain of 100%. The coil structural sensors monitor the bending angle of the finger (Figure 1a). Dong et al. [42] used polytetrafluoroethylene (PTFE) and a liquid metal alloy to prepare a stretchable microstructure fiber through the thermal drawing process. Regardless of repeated large deformations, the strains of the fiber could be stretched up to 560%. The fibers woven into the clothes act as a sensor to monitor breathing and the bending of the hand (Figure 1b). Furthermore, they weaved the fibers into deformable textiles, the electrical output signal of which could reach 490 V and 175 nC. Zheng et al. [43] prepared liquid metal (LM) sheath–core microfibers through the coaxial wet-spinning process. The maximum strain of the microfibers could reach 1170%. The glove woven by the LM sheath–core microfibers can monitor the activities of the finger and the wrist. Chen et al. [44] reported super-elastic fibers created using a two-step soluble-core fabrication method to form a polyvinyl alcohol (PVA) core/styrene–ethylene–butylene–styrene (SEBS) shell platform. Even in the case of 1900% strain, the ultra-stretchable fiber could maintain good conductivity. Combined with triboelectric nanogenerator technology, these self-powered multifunctional sensors can monitor sports performance. They constructed a fiber-based sensing net attached to a baseball glove’s inner surface, where it could locate hit points with different capture speeds (Figure 1c).

(2) Flat-shaped structure. Although the axial stretchability of flat-shaped flexible sensors is not as good as that of single-fiber-shaped structures, flat-shaped sensors have multidirectional stretchability. Wang’s research group [45] fabricated a TENG with double-layer rubber that can produce triboelectric charge because of the inhomogeneous strain of the rubber. The upper layer was a network of Ag nanowires, and the lower layer was a Ni macro-porous foam-like structure. When the strain was 100%, it could maintain good stability within 1300 cycles. This is the first time that a double-layer silicone rubber structure (DS-TENG) was used to characterize the three-dimensional deformation of muscles. It reflects the deformation process of different muscle areas by monitoring the output voltage change (Figure 1d). Lu et al. [46] used several single small-sized TENG units on a rubber substrate to form a stretchable, flexible triboelectric nanogenerator with a laddered shape. It exhibited excellent performance under the strain of 120% and curvature of 90°. The sensor can monitor hand gestures and the motion state of the human (Figure 1e). Guo et al. [47] used low-temperature vulcanized silicon rubber and used hydrogen bonding crosslinking among polyethylene oxide (PEO), waterborne polyurethane (WPU), and phytic acid (PA) to fabricate the current collector (Figure 1f). The sensor could stretch up to 318%. It had high electrical output with an open-circuit voltage of 197 V. The patch could maintain good stability and be used as E-skin to effectively monitor different forms of force. Cheng et al. [48] used an inorganic modified composite film (polydimethylsiloxane (PDMS) and organo-montmorillonite-cetyltrimethylammonium bromide (OMMT-CTAB)) and ZnO nanowire prepared via the vapor–liquid–solid growth process to fabricate a stretchable triboelectric nanogenerator. The maximum strain was up to 580%. Its open-circuit voltage and short-circuit current could reach 160 V and 12.4 μA. It showed high sensitivity in dynamic force, temperature, and position detection. These sensitive functions allow a wide range of applications in smart robotics and touch screens. Zhang et al. [49] used conductive carbon black (CB, stretchable layer) and dielectric thermoplastic polyurethane (TPU, triboelectric layer) to fabricate a CT-TENG. The maximum strain was up to 646%. Through corona charging, the open-circuit voltage could reach up to 41 V. This sensor can monitor the physical signal of human motions, such as wrist bending and finger tapping.

It can be seen from Table 1 that, whether it is a single-fiber-shaped structure or a flat-shaped structure, the self-powered flexible sensor exhibits excellent scalability and output performance, which significantly improves its scope of application.

### 2.2. Self-Healing

Human skin, as the largest organ in the human body, perceives the external environment (pressure, temperature, etc.) and serves as a barrier to protect the body. However, more importantly, the human skin possesses intrinsic self-healing ability to fully recover from physical or mechanical damage by triggering inherent repair mechanisms to alleviate or avert injure and ultimately maintain the integrity of structure and function [55,56]. Generally, wearable devices, especially self-powered sensors, are readily scratched and/or mechanically cut due to inevitable internal and/or external damage (e.g., cracks formed by bending, stretching, and friction). Accordingly, various natural and biomimetic materials with self-healing function have been integrated into self-powered sensors to improve their robustness, stability, service life, safety, and reliability.

**Figure 2:**
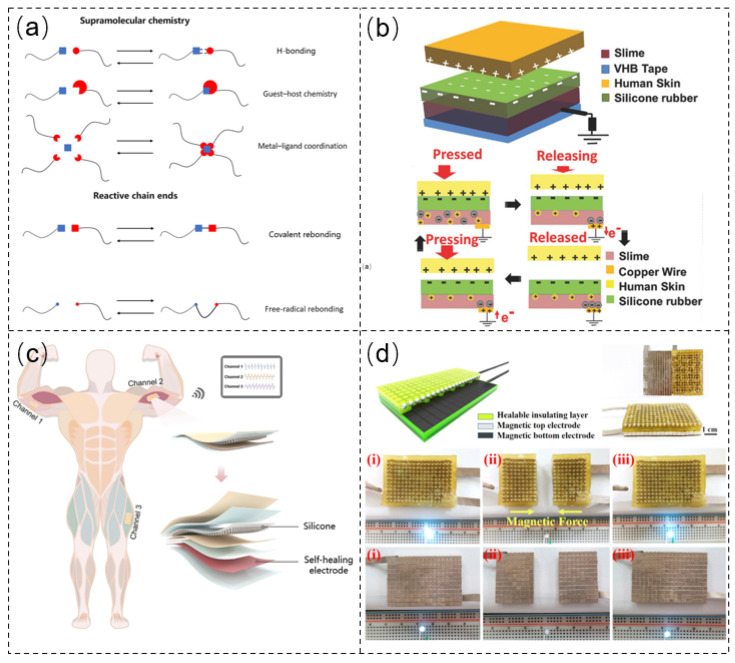
(**a**) Self-healing mechanisms via chemical approaches. (**b**) Self-healing slime-based ionic-skin TENG. Reprinted with permission from Ref. [57]. Copyright 2017 John Wiley and Sons. (**c**) Self-healing TENG based on an ionic hydrogel (PVA/PEI/LiCl) electrode. Reprinted with permission from Ref. [58]. Copyright 2021 American Chemical Society. (**d**) Completely self-healing TENG integrating a healable PDMS–PU elastic layer and magnetic electrode layer Reprinted with permission from Ref. [59]. Copyright 2017 Elsevier.

Conventionally, most existing self-healing materials are polymers (including natural and synthetic), and various physical, chemical, and even physicochemical approaches have been used to establish self-healing systems. By effectively constructing dynamic reversible intermolecular interactions, chemical approaches have been widely adopted to promote self-repair, including introducing reactive chain ends and supramolecular chemistry (Figure 2a). Therein, hydrogen bonds are the most widely adopted due to the advantages of directionality, moderate strength, and short healing time.

Recently, Parida et al. [57] demonstrated a spontaneous healing slime-based ionic-skin TENG featuring dynamic hydrogen bonds of the ionic conductor (as the current collector), which could restore its initial energy-harvesting performance even after 300 cycles of repeated mechanical damage. Despite the silicone rubber layer suffering mechanical damage, the surface charges remained the same as those of the nondamaged silicone rubber. Thus, the silicone rubber did not significantly influence the capability of the whole device due to the presence of the self-healing current collector (Figure 2b). Wang et al. [58] proposed a self-healing TENG utilizing an ionic hydrogel (PVA/poly(ethylene imine) (PEI)/LiCl) electrode based on the entanglement between polymer chains and dynamic hydrogen bonds to overcome the vulnerability of traditional metallic electrodes, whereby the whole structure and performance of the hydrogel could be quickly restored. After 10 min of self-healing, the wound coalesced wholly and vanished within 30 min at ambient temperature. Moreover, the maximum output of the assembled TENG could reach 78.44 V, while the output remained steady even after 10 cycles of damaging and self-healing processes (Figure 2c).

Most self-powered sensors usually employ two idiosyncratic materials to constitute the elastic layer (piezoelectric and triboelectric materials) and electrode layer (metal and conductive hydrogel). Thus, it is still urgent to realize complete self-healing of fundamental components after fracture. Accordingly, Xu et al. [59] fabricated a fully self-healing TENG by integrating a healable polydimethylsiloxane–polyurethane (PDMS–PU) elastic layer and an innovative healable magnetic electrode layer. The self-healing ability of PU–PDMS was ascribed to the introduction of disulfide links and multiple dynamic hydrogen bonds. Accordingly, based on the synergetic mechanism, both the open-circuit voltage and the short-circuit current of the constructed device could recover >95% of the initial values even after the fifth damage-healing cycle (Figure 2d). Thereafter, Xun et al. [60] developed a highly vigorous and self-powered E-skin with a low modulus misfit by constructing two self-healing polyurethanes (PUs) with similar structures. The outstanding self-healing power of the E-skin was realized by introducing disulfide bonds and multiple dynamic hydrogen bonds. When cutting a groove on the insulated PU layer or the conductive PU layer, even at the interface between the insulated and conductive PU layer, the groove gradually vanished after 5 min and fully recovered its initial state after 1 day at room temperature. After fully self-healing, the E-skin not only recovered its structure but also efficiently restored its stretchability (92%) and sensing performance (71%).

To enhance the self-healing capability of self-powered sensors, self-healing processes feature hydrogen bonds and other interactions (such as disulfide bonds and metal-coordination bonds). For example, Han et al. [61] prepared a flexible and self-healing single-electrode TENG based on polyacrylic acid–gelatin–sodium chloride hydrogel (PAA–Gel–NaCl), which could rapidly self-heal within 2.5 min at room temperature. The excellent self-healing properties of the hydrogel were deemed to be the combined effect of the triple helix crosslinking of gelatin and the dynamic reversible crosslinking network formed by the dynamic hydrogen bond between PAA and Gel molecules. Jing et al. [62] reported an ultra-stretchable and self-healing composite hydrogel based on polyacrylic acid/nanochitin (PAA/NCT) by introducing dual crosslinked networks. Due to the presence of dynamically reversible metal coordination bonds and multiple dynamic hydrogen bonds, it gave the hydrogel outstanding self-healing efficiency (97%). When it was assembled into a TENG based on a single-electrode model, the device could be used not only as an efficient energy harvester, but also as a self-powered pressure sensor. Jiang et al. [63] reported a highly stretchable TENG based on self-healing PDMS–PU_x_–PA_1-x_–Zn (PU: polyurethane, PA: polyamide) elastomers, which could simultaneously and quickly self-heal after fracture and wear at room temperature. By incorporating dynamic metal–ligand coordination and hydrogen bonds into PDMS networks, the self-healing polymer possessed outstanding self-healing ability within 10 min at room temperature (100% efficiency). When the assembled TENG was working in contact-separation mode, the electrical outputs could reach 140 V, 40 nC, and 1.5 μA. Even when stretched to breaking point or scratched to introduce wear, it could recover its electrical outputs within 20 min and 2 h at room temperature, respectively.

Benefiting from the outstanding self-healing ability of advanced materials by introducing dynamic reversible interactions, self-powered sensors exhibit superb performance in applications, whether they serve as the power source or the whole system.

### 2.3. Degradability

Degradable materials used in self-powered sensors are widely investigated because of their green and recyclable properties. Since the birth of the world’s first cellulose paper-based piezoelectric nanogenerator (PENG) in 2011 [64], green and recyclable nanogenerators have entered people’s sight. Wang’s team was the first to use paper-based materials to prepare TENGs [65]. However, in this experiment, the paper-based material was only used as the inner layer spacer and was not used for the triboelectric layer.

The use of degradable materials to prepare self-powered sensors is based on paper-based recyclable self-powered sensors. Zheng et al. [66] reported for the first time the use of artificial degradable materials to prepare the triboelectric layer in self-powered sensors, and they proposed for the first time the concept of “degradable and implantable TENGs”, which extended the application of TENGs into the field of medical health monitoring and realized degradation.

At present, degradable materials used in self-powered sensors can be divided into three categories: animal-based degradable materials, plant-based degradable materials, and artificial degradable materials.

**Figure 3:**
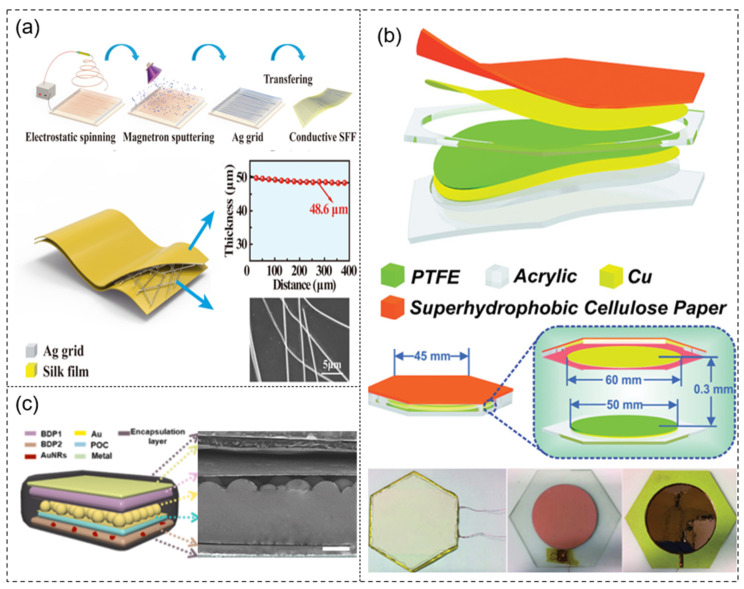
(**a**) Process and structure of TENGs made from animal-based materials. Reprinted with permission from Refs. [71,72,76]. Copyright 2020 Elsevier, Copyright 2021 American Chemical Society, Copyright 2018 Royal Society of Chemistry. (**b**) Basic structure scheme and digital photograph of plant-based materials. Reprinted with permission from Refs. [26,77,78]. Copyright 2018 Elsevier, Copyright 2014 John Wiley and Sons, Copyright 2020 John Wiley and Sons. (**c**) Structure design of TENGs made from artificially degradable materials. Reprinted from Ref. [66] and Reprinted with permission from Ref. [79]. Copyright 2018 Elsevier.

(1) Animal-based degradable materials. Currently, the most popular degradable materials used in self-powered sensors are animal-based materials. Among them, proteins and their derivatives, such as silk and egg white [67,68], can be used as the triboelectric layer of TENGs. These proteins can achieve rapid degradation in the environment of proteases and microorganisms while having good biocompatibility. In addition, the chitin fibers in the hard shells (crabs) of crustaceans are also degradable materials that can be used to prepare triboelectric layers [69,70]. At present, the preparation of biodegradable TENGs from animal-based materials is attracting more and more research attention [71,72,73,74]. Gong et al. [71] used a solution casting method to fabricate a conductive silk fibroin film (SFF), deposited a silver layer onto the PVA network by magnetron sputtering, and then transferred the Ag/PVA network to the SFF (Figure 3a). They developed a triboelectric nanogenerator (Bio-TENG) with high light transmittance, biocompatibility, biodegradability, softness, and flexibility. However, the chemical stability and fragility of the silk fibroin membrane limited its application. Xu et al. [72] studied the mesoscopic doping of regenerated silk fibroin to address the inherent brittleness and poor chemical stability of pure silk fibroin films. Their design can be used in self-powered mechanical sensor communication systems in smart cars. For a long time, the development of degradable PENGs was restricted by toxicity and high cost. Research on biodegradable PENGs has benefited from the preparation of TENG and PENG composite nanogenerators in recent years. Kim et al. [75] developed a biodegradable composite nanogenerator based on silk fibroin, which has a controllable lifespan and can power implantable devices. By using silk fibroin solution and lead-free ferroelectric nanoparticles, the composite material was made into two-dimensional thin films and one-dimensional wires. At present, there are few studies on degradable PENGs, and the degradable materials used in PENGs are mainly animal-based materials. Hoque et al. [76] reported two high-performance PENGs based on chitin nanofibers (CNFs) extracted from crab shells. They were pure CNF-based PENG and CNF/PVDF nanocomposite membrane-based devices. The biocompatible and biodegradable PENGs can be used in portable gadgets.

(2) Plant-based degradable materials. At present, self-powered sensors made from plant-based materials are regarded among the main representatives of future green electronic products and are considered to have great development prospects. In particular, PENGs and TENGs based on paper/cellulose materials are a new source of renewable energy with low cost and a rich source of raw materials, attracting the attention of many researchers [26,77,78,80,81]. Luca et al. [77] took the lead in using plant-based alginate and a GO composite film in a TENG, and a composite film was prepared (Figure 3b). However, the final result was far from the output power density obtained when using nanostructured TENGs. Paper is the most widely used material in plant-based degradable designs, and there have been many reports on paper-based PENGs and TENGs. Shi et al. [26] developed a flexible high-performance PENG using regenerated cellulose/BaTiO_3_ (C/BT) aerogel paper based on polydimethylsiloxane (PDMS) nanocomposites. Nie et al. [78] developed a new type of cellulose paper-based D-TENG, which had strong, self-cleaning, and super-hydrophobic properties and could harvest energy from raindrops. The honeycomb water droplet energy-harvesting device can power computers and calculators and can also be used on the roof.

(3) Artificial degradable materials. Artificial degradable materials synthesized by various methods have played an important role in self-powered sensors [82,83]. Recently, there have been some reports on the application of artificially degradable materials to TENGs. As mentioned above, Zheng et al. [66] first proposed the use of artificially degradable materials to prepare the triboelectric layer in self-powered sensors.. The triboelectric layer was made from different degradable materials and encapsulated with poly(l-lactide-*co*-glycolide) (PLGA) or PVA (Figure 3c). The study observed the degradation of BD-TENG in vitro and found that it could be completely degraded within 90 days. Subsequently, Li et al. [79] used artificially degradable materials together with gold nanorods to prepare a triboelectric layer, and proposed for the first time the use of NIR light to adjust the degradation rate of the TENG implanted in the body. The triboelectric layer was made of different degradable materials. Biodegradable polymer (BDP) doped with AuNRs was used as the bottom substrate, and the other layer of magnesium was deposited as the top electrode. The top and bottom parts were sealed with BDP solution. The study evaluated the in vitro degradation of various BD-iTENGs (size, 1.2 cm × 1.2 cm; thickness, 0.65 mm) under different conditions.

It can be shown from Table 2 that, when artificially degradable materials are used to prepare the triboelectric layer, the value of the open-circuit voltage is larger, which can exceed 20 V. On the other hand, when animal-based degradable materials are used to prepare the triboelectric layer, the open-circuit voltage and short-circuit current are also relatively large.

### 2.4. Multifunction

**Figure 4:**
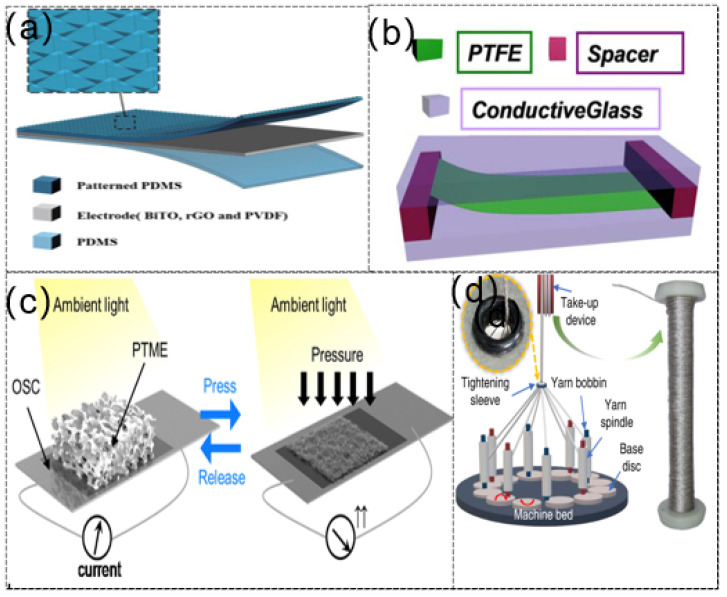
(**a**) Schematic illustration showing the basic structure of tactile e-skin. Reprinted with permission from Ref. [85]. Copyright 2020 Elsevier. (**b**) Schematic diagram of ATNG sensor. Reprinted with permission from Ref. [86]. Copyright 2014 American Chemical Society. (**c**) Schematic diagram of the principle of PTSP and OSC sensors. Reprinted with permission from Ref. [87]. Copyright 2020 Elsevier. (**d**) Schematic diagram of multi-shaft yarn winding machine. The enlarged picture in the upper left corner is a picture of the tightening sleeve and the bobbin wrapped with continuous multi-shaft winding yarn. Reprinted from Ref. [88].

Multifunctional flexible sensors, which can effectively solve the problem of the limited application range of single-performance sensors, have been widely studied in recent years [84]. Rao et al. [85] proposed a sensor which can simultaneously detect pressure and temperature, composed of a thermosensitive electrode combining rGO and BiTO; the sensor is suitable for tactile e-skin (Figure 4a). The tactile e-skin offers new routes for wearable sensing and provides new insights for areas such as medical care and robotics (Figure 4b). Guo et al. [86] designed a novel humidity sensor that could be used in monitoring humidity via an airflow-induced triboelectric nanogenerator (Figure 4c). The novel device is usually used in air humidity and flow rate monitoring. The PTFE film vibrates when the air flows through the airflow-induced triboelectric nanogenerator (ATNG), and the PTFE film constantly vibrates up and down with the air and rubs with two electrodes becoming charged, determining the airflow rate according to different output currents. The experimental results showed that, when the external environment RH (relative humidity) was less than 80%, the air flow was correlated with the output current. The study suggested that these ATNG devices can be used as self-powered sensors in air quality inspection. Choi et al. [87] fabricated a unique wearable self-powered pressure sensor combining a piezo-transmittance microporous elastomer (PTME) and a thin-film organic solar cell (OSC). The PTME micropores gradually closed with the change in applied pressure and light transmittance after compression. This special optical property of PTME enabled the OSC to respond to the current changes under pressure. This design was applied to robot prosthetic finger sensing. The motion of the human finger is detected, and the signal is fed back to manipulate the prosthetic robot finger to perform the same motion.

Traditional sensors have defects such as unstable performance and poor sensing capabilities. Moreover, most TENGs have a planar structure, occupy an ample space, and are very unsightly and inconvenient in wearable applications. Dong et al. [88] reported a TENG-based e-textile designed with a three-dimensional five direction braid (3dB) structure (Figure 4d). Under 3 Hz loading frequency and 20 N force, the device’s open-circuit voltage could reach 90 V and the peak power density could reach 26 W/m^3^. The excellent structure enabled 3dB-TENG to have shape adaptability and corresponding slight weight changes. It was applied to an intelligent running shoe insole for human motion monitoring and a carpet that automatically identifies identity. It can see from Table 3, lots of factors can influence the properties of self-powered multifunctional flexible sensor.

A TENG’s performance is greatly affected by the environment’s humidity. Liu et al. [89] proposed a strategy to enhance the output performance of TENGs in a high-humidity environment through coupling dielectric material selection and surface-charge engineering. The output performance of the PTFE-based TENG was more stable in a high-humidity environment, whereby the device charge density could retain ~97% of the initial value. When it underwent 4500 cycles, the same was true at high temperatures. In a high-humidity environment, the electrical output performance of the TENG based on ion implantation on the surface of polytetrafluoroethylene (PTFE) was much higher than that of other TENG devices.

**Table 3:** Comparisons of self-powered multifunctional flexible sensor.

Composition of Sensor	Function	Output Performance	Sensitivity	Influence Factor of Sensitivity	Mechanism of Sensor
rGO, BiTO, PVDF, PDMS	Temperature, pressure	/	1024 K, 5.07 mV/Pa	pyramidal microstructures	TENG
FTO glass, PTFE	Humidity, airflow rate	38 V, 4.2 μA	/	/	TENG
PTME, OSC	Pressure, wind speed and direction	/	0.101/kPa	Optically active structures	Piezo-transmittance
Multiaxial winding yarn, energy yarn	Human motion monitoring, safeguarding entrance and identity information	90 V, 26 W/m^3^	/	/	TENG

## 3. Structural Design

### 3.1. Principle and Structure of Nanogenerators

#### 3.1.1. Principle and Structure of PENGs

Conventionally, PENGs consist of an insulation as piezoelectric layer sandwiched between two conductors [90]. Inorganic piezoelectric materials cannot be directly applied in the field of flexible electronic skin due to their brittleness [91]. PVDF and its copolymers have better piezoelectric properties. At present, there are many kinds of inorganic materials such as barium titanate (BaTiO_3_), zinc oxide (ZnO), and flexible polymer materials that are usually used to prepare high-performance piezoelectric materials. PVDF and its copolymers can be compounded to improve its polarization and obtain high-performance piezoelectric materials by using inorganic nanomaterials as nucleating agents.

**Figure 5:**
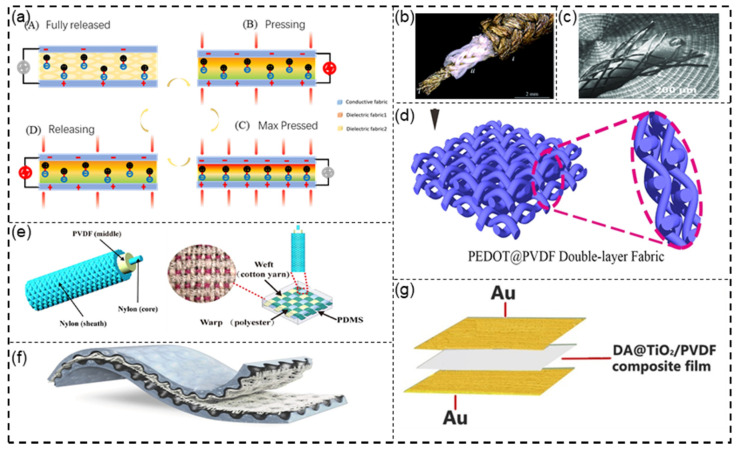
(**a**) Working mechanisms of textile-based PENGs. (**b**) One-dimensional single fiber-based PENG Reprinted with permission from Ref. [96]. Copyright 2019 Royal Society of Chemistry. (**c**) One-dimensional single fiber-based PENG Reprinted with permission from Ref. [97]. Copyright 2020 John Wiley and Sons. (**d**) Two-dimensional fabric-based PENG with textile forming structures. Reprinted from Ref. [98]. (**e**) Two-dimensional fabric-based PENG with textile forming structures Reprinted with permission from Ref. [99]. Copyright 2021 John Wiley and Sons. (**f**) Three-dimensional fabric-based PENG with multilayer stacking structures Reprinted with permission from Ref. [100]. Copyright 2019 John Wiley and Sons. (**g**) Three-dimensional fabric-based PENG with multilayer stacking structures Reprinted with permission from Ref. [101]. Copyright 2021 Elsevier.

We detailed a complete electrical generation process for a PENG during one pressing and releasing process (Figure 5a). In the beginning, the centers of positive and negative charges in the piezoelectric layer coincide with one another. Thus, current generation cannot be observed in the external circuit. The piezoelectric layer will deform when a pressure load is applied, resulting in a decrease in thickness, and the internal charge center is separated to form electric dipoles and a change in electric dipole moments. An electric potential is formed between the two electrodes, and electron transfer occurs in the external circuit to achieve a new equilibrium. In this process, mechanical energy is converted into electrical energy. The balance of charges between the piezoelectric layer and the electrodes leads to current in the external circuit disappearing when the maximum pressure is reached. When the external force is released, the centers of cations and anions in the piezoelectric layer gradually become overlapped again. The charges between the electrodes flow back through the external circuit and rebalance. The piezoelectric layer returns to its original thickness, and the current in the external circuit disappears with the external force fully released (Figure 5a). A pulsed current can be formed in the external circuit by continuously applying and releasing pressure.

At present, many attempts have been made to simultaneously improve the flexibility and power output performance of nanogenerators [92,93,94,95]. They can be divided into three types according to weaving methods and structural forms: 1D single-fiber-based PENG, 2D fabric-based PENG with textile forming structures, and 3D fabric-based PENG with multilayer stacking structures.

(1) 1D single-fiber-based PENG. The 1D single-fiber-based PENG is the simplest structure of textile nanogenerators, which can be directly fabricated into 2D and 3D PENGs. In recent years, many scholars conducted research and achieved good results. Mokhtari et al. [96] fabricated a PENG (Figure 5b) in a structure with melt-spun PVDF filaments braided around silver-coated nylon yarns. The measurement results showed that the peak value of output voltage was 380 mV and the power density of 29.62 μW/cm^3^ could be produced by compressing or bending the single-fiber nanogenerator, which was about 1559% higher than previously reported piezoelectric textiles. Razavi et al. [97] fabricated another single-fiber nanogenerator (Figure 5c). This structure featured a number of PVDF yarns braided around copper and coated by copper wires; it achieved excellent mechanical performance and piezoelectric properties. They found that the mechanical performance and power output were strongly determined by the structure of the nanogenerator as a function of changing the number of PVDF yarns and outer copper wires.

(2) 2D fabric-based PENG with textile forming structures. Although the simple structure of 1D single-fiber-based PENGs brings great convenience for manufacturing, its electromechanical conversion efficiency and electric energy output do not meet expectations due to the limitations of size and structure. In order to obtain the desired electrical power output, an efficient method is to weave 1D fibers into 2D or 3D fabrics. Zhou et al. [98] reported a highly sensitive, self-powered, and wearable electronic skin through weaving PVDF electrostatic spinning yarns of nanofibers coated with poly(3,4-ethylenedioxythiophene) (PEDOT) (Figure 5d). Its sensitivity, test pressure range, response time, and durability all showed excellent performance. Devices made from this form of fabric are successfully used in monitoring muscle movement on the human face, monitoring voices during singing, and monitoring current changes according to the wrist pulse before and after exercise. Xue et al. [99] fabricated a 2D fabric-based PENG through braiding 1D structure nanogenerators with PVDF electrospun yarns around a conductive nylon core and coated by outer conductive nylon yarns (Figure 5e). The fabric was complex in that the warp was made from polymers, and the weft was composed of a single-fiber PENG and cotton yarns. The purpose of cotton yarns was to avoid short-circuits in generators.

(3) 3D fabric-based PENG with multilayer stacking structures. Fabric-based PENGs can be prepared in the form of traditional textiles by stacking two-dimensional textile structures. A stacked nanofiber mat was alternatively composed of nanocomposite nanofibers of BaTiO_3_ nanoparticles embedded in PU and polyvinylidene fluoride–trifluoroethylene copolymer (PVDF–TrFE) nanofibers (Figure 5f). The surfaces of the piezoelectric layer were coated with a stretchable graphite electrode. Then, the device was based on PDMS as substrate [100]. This multilayer nanogenerator exhibited a high stretchability of 40%, and it could be recycled 9000 times under 30% strain. These properties were attributed to its nanostructure and variability of the electrodes. Liu et al. [101] fabricated a multilayer PENG, constituting a composite of dopamine (DA)-coated TiO_2_ and PVDF as the piezoelectric layer and Au as the electrode (Figure 5g). Furthermore, DA2.0 @TiO_2_/PVDF with a 84.33% β-phase was successfully fabricated via the solvothermal method. The piezoelectric coefficients and remnant polarization of the DA2.0 @TiO_2_/PVDF were 163% and 278% higher than those of the pure PVDF film.

PENGs can only convert pressure into electrical energy; however, in everyday life, substantial power is lost because it cannot be collected, such as friction energy. In recent years, scholars have focused more and more on the research of devices that can convert friction energy into electrical energy [102,103,104]. Accordingly, TENGs can be used not only for self-powered sensors but also as wearable devices.

#### 3.1.2. Principle and Structure of TENGs

A triboelectric effect exists between any two objects, even if they are of the same material. There are cations and anions that appear when two different materials or irregular surfaces of the same material come into contact. In addition, contact between materials at two different temperatures also produces the triboelectric effect, whereby the hotter one tends to be positively charged, while the cooler one tends to be negatively charged [105]. Therefore, the selection of TENG materials is very extensive; we usually refer to the charged sequence when choosing the dielectric material.

**Figure 6:**
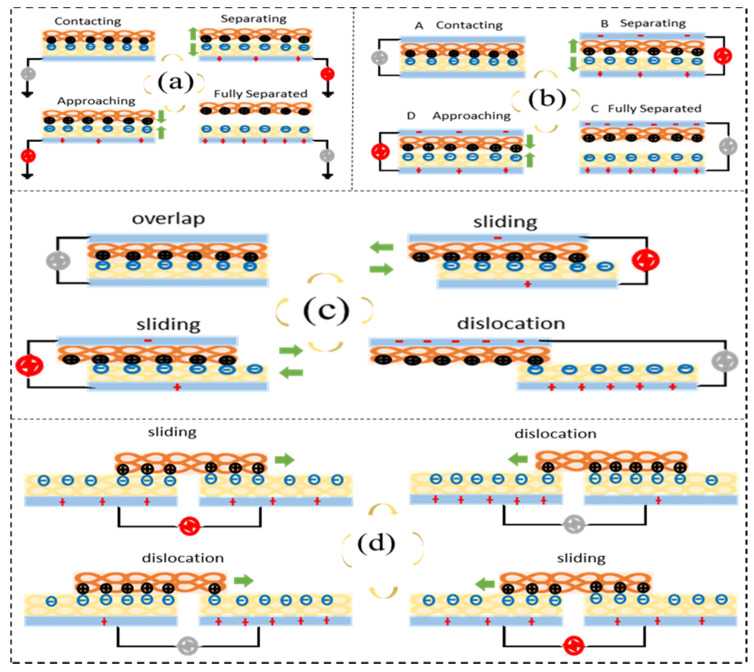
Working mechanisms of textile-based PENGs. (**a**) Single-electrode model. (**b**) Vertical contact-separation model. (**c**) Lateral-sliding model. (**d**) Freestanding triboelectric-layer model.

TENGs can be divided into four models: the single-electrode model, lateral-sliding model, vertical contact-separation model, and freestanding triboelectric-layer model (Figure 6). Although they come in different forms, they all work on the basis of charged effects and electrostatic induction. The single-electrode model (SE) only has one electrode and takes the ground as the reference electrode (Figure 6a). The lateral-sliding model (LS) relies on the relative motion of the contact surfaces to generate an electric current (Figure 6b). The vertical contact-separation model (CS) generates current in the external circuit through contact and separation motion vertical to the contact surface (Figure 6c). The freestanding triboelectric-layer model (FT) has a dielectric layer that can move freely (Figure 6d).

Since the four models have the same working mechanism, the CS model is used below as the representative to introduce TENG design. Firstly, the dielectric materials of the upper and lower layers touch each other. They generate opposite electrical charges at the contact surface and balance each other such that there is no current in the external circuit (Figure 6b). When the upper and lower layers of the dielectric material are separated, the positive and negative charges that are balanced on the contact surface are separated on both sides, and electrons need to be transferred between the electrodes via an external circuit to achieve a new balance, thus generating a current. When the upper and lower dielectric layers are completely separated, the entire nanogenerator is rebalanced and the current disappears. On the contrary, when the upper and lower two-layer dielectric materials are close to each other, the electrons on the electrode flow back through the external circuit to form the current. The positive and negative charges reach a new balance in the contact layer, and the current in the external circuit disappears when the two dielectric layers are in full contact.

Like PENG, TENG can be divided into three types according to shape and structure: 1D single-fiber-based TENG, 2D fabric-based TENG with textile forming structures, and 3D fabric-based TENG structures.

Whether the structure is complex or not, its most basic principle involves a charged effect and electrostatic induction. A single fiber/yarn is the smallest design unit for textile-based TENGs. One-dimensional single-fiber-based TENGs are widely designed and applied due to their excellent performance and simple structure. Zhang et al. [106] reported an electronic yarn twisted into a Fermat spiral with outstanding performance and dynamic structure stability. The single-fiber-based TENG consisted of spandex yarn as a flexible core, braided conductive fibers as the electrode, and a PVDF–TrFE braided outer as the dielectric. This typical single-electrode model relies on axial friction between the dielectric layer and conductive fiber to generate electricity. It was proven to have ultrahigh stability and excellent electrical output performance. It can be used for a variety of complex energy acquisition and motion perception applications, such as gesture recognition and droplet power generation. Han et al. [107] developed a multifunctional coaxial energy fiber, which integrated energy collection (TENG), energy storage (a supercapacitor), and sensing (pressure sensor). The inner core was an energy collector formed by PVA/H_2_SO_4_ wrapped around carbon fiber, and the outer sheath was fibrous. The design constituted a self-powered pressure sensor. In order to improve the power output, as well as promote their wide application, textile-based TENGs usually exist in the form of fabric. Conductive or dielectric fibers can be processed into fabrics via various textile forming techniques. Cong et al. [108] built an FT-model TENG using nickel fibers as electrodes, PDMS coated with Ni fibers, and other fabrics as dielectric materials. Although flexible nanogenerators have good deformation properties, their resistivity is far from ideal compared with metal, which undermines their potential for practical applications. Jing et al. [109] applied the basic physical concepts of a small resistance in parallel circuits to triboelectric nanogenerator fibers/textiles to reduce the negative effects of large resistance in extendable electrode fibers. The results showed that the parallel structure significantly reduced the resistance and increased the output power 11.8-fold. Two-dimensional textiles are popular in intelligent textile design because of their relatively simple structure, convenient preparation, and compatibility with existing textile processing technology. However, due to the limitation of structure size in the thickness direction, the power output of traditional 2D textiles is still low. In order to further improve the output performance of electric textiles, 3D textile structures have been gradually adopted. Zheng et al. [110] developed a single-electrode TENG with 3D structure, constituting PTFE and Pb(Zr, Ti)O_3_ (PZT)/glass fiber fabric as the dielectric layers and Altium as an electrode. The generator had excellent output performance and could simultaneously light up to 1350 LEDS. Wang et al. [111] created a three-dimensional TENG whose electrodes were silver-coated nylon fibers, which were braided with PE and PTFE and then woven into a two-dimensional fabric as dielectric layers. The dielectric layers were separated by other fibers to ensure a greater range of movement and, thus, greater power output. The device, which can monitor a person’s sitting and walking state, provides a new 3D intelligent textile structure that may facilitate the application of TENGs in wearable micro/nano power supplies, self-powered sensors, and human–machine interfaces.

### 3.2. Bionic Structure

**Figure 7:**
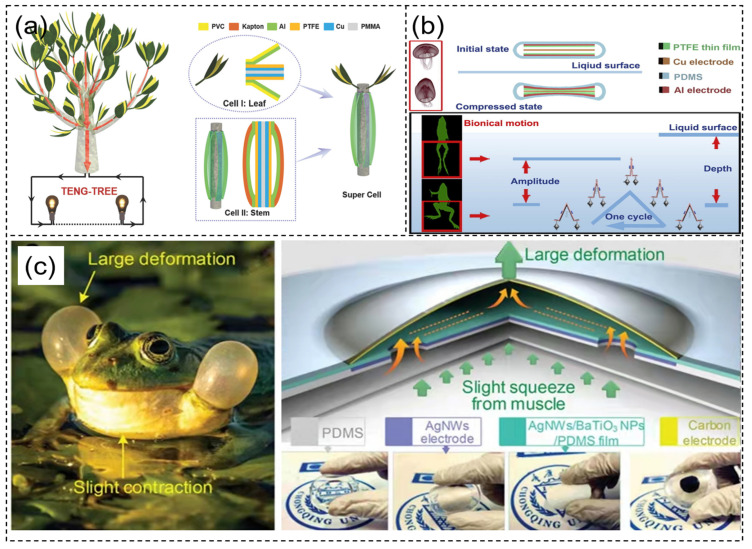
Several bionic structures. (**a**) Working principle of bionic leaf structure and bionic stem structure. Reprinted with permission from Ref. [115]. Copyright 2018 John Wiley and Sons. (**b**) Working principle of bionic jellyfish structure. Reprinted with permission from Ref. [116]. Copyright 2017 Elsevier. (**c**) Working principle of the bionic structure reflecting morphology of the frog when it croaks. Reprinted from Ref. [119].

Many organisms in nature feature an optimal shape following thousands of years of evolutionary history; hence, mimicking the structure of organisms to design nanogenerators might achieve better performance. In recent years, more and more scholars have begun studying bionic nanogenerator structures [112,113,114]. Bian et al. [115] designed a bionic nanogenerator that harvests wind energy, modeled on leaves and stems. In the bionic leaf structure, aluminum and PTFE were used as dielectric layers. The aluminum of the dielectric layer was also used together with copper as the electrode layer, and the upper and lower surfaces were covered with polyvinyl chloride (PVC) and polymethyl methacrylate (PMMA), respectively (Figure 7a). Four supercells were connected in parallel to form a TENG tree; the output voltage and current could reach 330 V and 59.6 µA at the wind speed of 11 m/s. Using this compound TENG, they designed a device to collect wind energy generated by a moving subway passing through a tunnel to illuminate the lights and billboards inside the tunnel. Chen et al. [116] modeled a TENG after a jellyfish that can deform freely on the basis of the contact-separation mode. It was a symmetrical structure that worked under the pressure of the sea. The dielectric layer was made up of PTFE film and copper, and the electrode was made up of aluminum on both sides and copper in the middle; the overall structure was packaged with PDMS (Figure 7b). This TENG can be used as a power source for self-powered LED lighting systems in applications such as lighting and coastal navigation warnings. Li et al. [117] developed a PENG by constructing bionic ion channels. The piezoelectric layer consisted of a PVDF membrane with bionic ion channels filled with ionic liquid, as well as a carbon composite covered with copper foil as electrodes on both sides, overall encapsulated with polyethylene terephthalate (PET). The composite nanogenerator could achieve an output surface charge density of up to 24.5 mC/m^2^ and a short-circuit current of 13.3 µA. At a low frequency pressure of 1 Hz, the device could obtain an open-circuit voltage of 150 mV within 80 s. Connecting multiple such devices in series can be used to power small wearable electronic devices. Li et al. [118] proposed a self-cleaning and water-resistant leaf-like bionic structure TENG modeled on the lotus leaf shape. The lotus texture was formed on the surface of the device using the template method, granting the device excellent waterproof and self-cleaning ability. This friction nanogenerator can be used to capture the energy of wind and raindrops in the natural environment. Zhou et al. [119], inspired by frogs’ croaking behavior, a developed a bionic TENG. The TENG consisted of a bottom electrode based on Ag NWs, a composite triboelectric layer based on Ag NWs/BaTiO_3_NPs/PDMS, and a top electrode based on carbon. The bottom electrode and triboelectric layer were integrated as a spacer (Figure 7c). This working principle of the bionic structure reflects the morphology of the frog when it croaks. The sensor had good sensitivity and intensity with respect to its signal sensing range. Its intensity was 206 times that of traditional biopotential electromyography.

Complete imitation of biological movement is not required in most cases; rather, a combination of biological and TENG techniques should be used to construct the components. Therefore, TENGs have ample room for new structural designs inspired by nature.

### 3.3. Origami and Kirigami Structure

The critical issue for traditional TENGs when applied in energy-harvesting devices and flexible wearable devices is to develop adaptive, simple-structured, high-performance but low-cost TENGs for the complex excitation conditions. In order to solve this problem, many researchers have sought breakthroughs using origami and kirigami structures in recent years.

**Figure 8:**
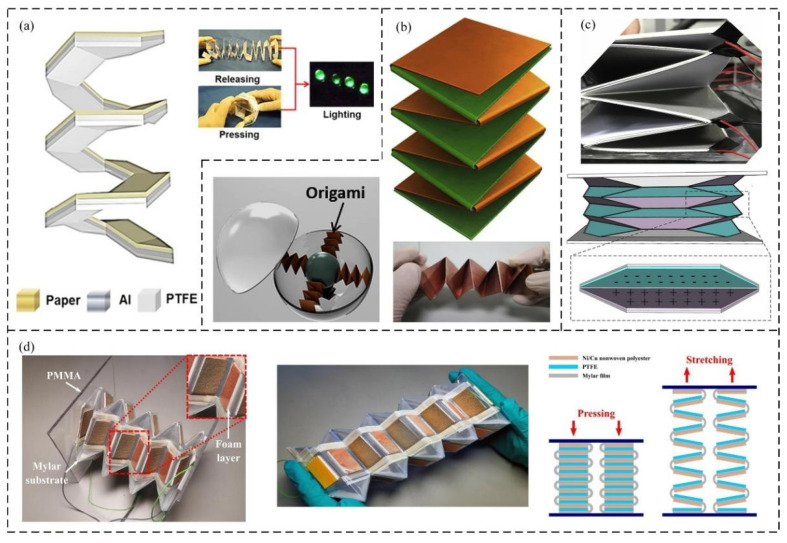
Multiple origami-type TENGs. (**a**) Schematic diagram and physical image of spiral paper-based origami TENG. Reprinted with permission from Ref. [120]. Copyright 2015 American Chemical Society. (**b**) Double-helix structure of stretchable TENG pressure sensor. Reprinted with permission from Ref. [121]. Copyright 2019 Elsevier. (**c**) Origami TENG applied in the field of intelligent transportation. Reprinted with permission from Ref. [122]. Copyright 2020 Elsevier. (**d**) TENG with polyester film based on origami structure. Reprinted with permission from Ref. [123]. Copyright 2021 Elsevier.

Origami-type TENGs, which are usually designed as layered structures, improve the output performance of the device by increasing the number of friction layers and improving the acceptance rate of mechanical behavior. In recent years, researchers have developed various types of origami structures according to different application requirements. For instance, a stretchable TENG pressure sensor with a spiral paper-based origami structure was reported in [120]. This paper-based origami TENG was composed of three layers of materials. During the compression process, the spirally folded structure enables the upper surface electrodes of each layer to contact the lower surface electrodes, generating electric charges. Moreover, application experiments showed that the manufactured TENG can harvest ambient mechanical energy from various kinds of human body movements, such as stretching, compression, and twisting. Therefore, it can be applied to human health and exercise monitoring (Figure 8a).

In another design, a double-helix origami structure was proposed to further increase the structural flexibility and energy conversion rate [121]. The double-helix spring-like origami architecture endowed the proposed TENG with excellent elastic and self-rebounding properties without any auxiliary resilient support, making the whole device compact, lightweight, and extremely sensitive. On the other hand, with its unique origami multifold structure and double-sided corona discharging process, the electric charge density and electrostatic induction were significantly enhanced. Due to its lighter weight and excellent flexibility, this double-helix origami TENG shows great potential in smart wear and ocean wave signal acquisition applications (Figure 8b).

In addition to being affected by the overall device structure, the electrical output characteristics of current origami nanogenerators are also limited by the simple origami patterns used as the basis of TENGs. In response to this problem, an origami tessellation (OT) base was reported [122]. This OT base can be installed with multilayer friction pairs and can be driven by very little stimulation. Moreover, the increase in paper-based tribo-pairs could significantly enhance the electric output performance of the device. The application demonstration showed that OT-TENG has potential in the future energy demands of intelligent transportation (Figure 8c).

Furthermore, researchers recently made a flexible and lightweight origami TENG using conductive Ni/Cu nonwoven polyester and Mylar film [123]. The support and rebound effect of the Mylar film could eliminate the additional auxiliary support system, leading to better stability of the TENG, along with electrostatic induction and triboelectric contact. Subsequently, a self-powered control power management circuit was designed to maximize the power delivered to the electrical load (Figure 8d).

**Figure 9:**
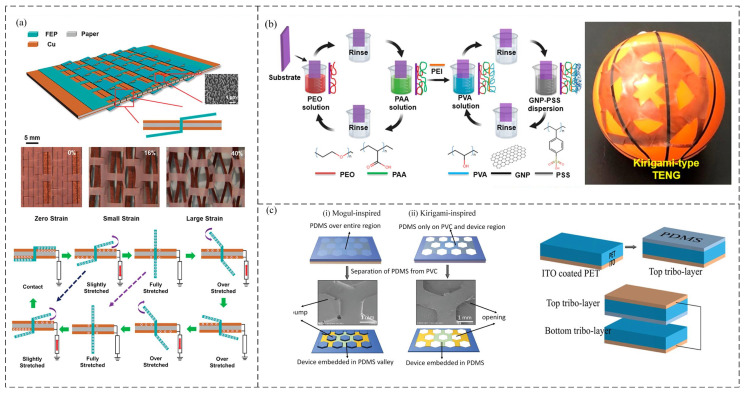
TENGs with kirigami structure. (**a**) Stretchable TENG with grid kirigami structure. Reprinted with permission from Ref. [124]. Copyright 2016 American Chemical Society. (**b**) Kirigami TENG with super stretching ability and extraordinary folding ability. Reprinted with permission from Ref. [125]. Copyright 2021 American Chemical Society. (**c**) Kirigami TENG with wearable photodetector function. Reprinted from Ref. [126].

In addition to origami structures, kirigami is used to design and develop new types of nanogenerators. The introduction of kirigami TENG (KTENG) structures can improve the energy collection efficiency and stretchability of the device. A highly stretchable, environmentally friendly paper-based TENG with rationally designed interlocking kirigami structures was reported in [124]. The interlocking structure allows the TENG made of inherently nonelastic materials to withstand ultrahigh tensile strains of up to 100%. The kirigami structure design eliminates the separation interval of traditional TENGs. Owing to this shape-adaptive thin-film design, the KTENG can harvest environment energy from various types of motion (stretching, pressing, and twisting) (Figure 9b).

Furthermore, the kirigami structure is also used for TENGs in electronic skin. Dukhyun et al. [126] reported an ultrathin and stretchable acrylic acid ([PEO/PAA]n) ethylene oxide and double-layer positive triboelectric film, which was manufactured using a low-cost and environmentally friendly layer-by-layer method. The composite film was used to design a shape-adaptive kirigami nanogenerator. The excellent elasticity of the composite film and the design of the kirigami structure enabled the TENG to exhibit 900% super stretchability and extraordinary foldability (Figure 9b).

In addition, a polymer matrix with a kirigami structure can enhance the stretchability of a TENG. A self-powered stretchable wearable photodetector was developed using a kirigami-based honeycomb structure of zinc oxide nanowires and coupled with a TENG [125]. Embedding this device into a PDMS substrate with a kirigami pattern of honeycomb geometry showed outstanding stretchability with strains up to 125% (Figure 9c).

In addition, the merits of the Miura folding structure, such as light weight, large unfolding area, and small folding volume, have attracted interest for TENG applications. Recently, a novel charge-excitation TENG based on a Miura folding structure was developed by integrating flexible PET (as a folding substrate with a double working side design: one side working as the main TENG (M-TENG) and the other side working as the excitation TENG (E-TENG)); the output performance was largely increased due to this unique structure design, which could increase the TENG working area and reduce its volume [127]. The kirigami structure can impart deformability to non-stretchable hard materials and realize the conversion between a 2D planar shape and 3D three-dimensional configuration. Combining flexible paper-cutting technology and self-healing conductive materials, as well as using the configuration transformation characteristics of kirigami technology, through the healing process of self-healing conductive materials, it is possible to realize the planar manufacturing of curved circuits and the conformal assembly of curved surfaces.

## 4. Frontier Applications

### 4.1. Electronic Skin and Wearable Device

Electronic skin (e-skin) is a flexible electronic device covering the surface of a machine or human body, where the system can give the carrier the ability to sense touch, pressure, temperature, and chemical or biological information. After granting robots the ability to perceive through electronic skin, the application scenarios of robots can be broadened, which includes many actions that require a high degree of interaction, such as caring for the elderly [127]. With the development of science and technology, more and more versatile and powerful electronic skins have been invented [128,129,130,131,132,133,134]. In the application field, self-powered sensors based on TENGs and PENGs generally use wireless contact through a signal amplification receiver to obtain the signal curve, which can be used to analyze their sensing performance through the electrical signal [134]. At present, electronic skin can be integrated into tactile sensors in a prosthesis such that the amputee can have a better experience.

**Figure 10:**
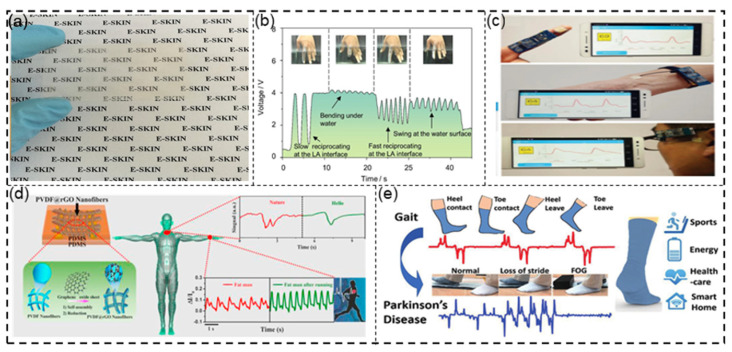
(**a**) Electronic skin using gold electrodes to simulate neuronal cells. Reprinted with permission from Ref. [129]. Copyright 2018 American Chemical Society. (**b**) Sketch of the S-LSNG attached to the fingers as a self-powered sensor. Reprinted with permission from Ref. [131]. Copyright 2020 Elsevier. (**c**) Highly sensitive graphene-based pressure sensors for applications as e-skins and wearable electronics. Reprinted with permission from Ref. [139]. Copyright 2016 Elsevier. (**d**) Flexible self-powered sensor that can be used to monitor gait. Reprinted with permission from Ref. [141]. Copyright 2019 John Wiley and Sons. (**e**) TPENG sock using PEDOT:PSS-coated textile. Reprinted with permission from Ref. [145]. Copyright 2019 American Chemical Society.

The emergence of self-powered devices such as PENGs and TENGs has significantly increased the application scenarios of electronic skin. Recently, many novel designs of electronic skins based on self-powered devices appeared. Wang et al. [129] used PVDF nanofibers to prepare a new type of single-electrode piezoelectric nanogenerator (SPENG), which can realize steady-state pressure sensing and integration on a single unit for cold/heat sensing. The sensor has excellent light transmittance and is very suitable to be worn on the human body (Figure 10a). Yao et al. [130] developed a bionic self-powered TENG electronic skin inspired by the surface morphology of natural plants. The team created interlocking microstructures on the friction layer to enhance the triboelectric effect. The ultrasensitive electronic skin can be connected to a manipulator to help the robot in terms of tactile perception and texture object recognition. In the same year, Liang et al. [131] produced a unique nanogenerator that can generate electricity by collecting the energy generated by small fluctuations and flow in the water, along with a waterproof function. By connecting the device to a human finger as a wearable device, the perception of finger movement in water can be realized. Its stretchable and ultrathin characteristics make it the best candidate for creating robot electronic skins.

In addition to their important role in the field of electronic skin, PENGs and TENGs also have many applications in wearable devices. PENGs and TENGs have brought about a revolution in wearable devices, replacing traditional rechargeable sensors and bringing great convenience to users. For example, in motion detection, the weight of the battery in the traditional wearable sensor is relatively large, which leads to inconvenience for the user. The use of self-powered sensors can effectively solve this problem and greatly reduce the overall equipment weight.

In the medical field, there are currently many reports on the use of self-powered sensors to detect pulse and respiration [135,136,137,138]. Lou et al. [139] prepared a flexible self-powered sensor based on a TENG and applied it to the human body, where it could stably measure the human pulse and blood pressure. It can be worn on people’s fingers, wrists, and ears for real-time pulse wave measurement (Figure 10c). Zhao et al. [140] prepared a completely transparent and highly stretched contact-separation TENG based on PDMS and used it for self-powered tactile sensing. In the case of different stretch ratios (0%, 10%, 50%, and 80% strain), the triboelectric signal maintained a good linear correlation, allowing the sensor to detect various human activities. It can sensitively respond to finger touches and bends and can detect breathing and pulse.

In terms of motion detection, wearable devices are mainly used to record the number of steps and monitor gait (Figure 10d) [141,142,143]. Zhu et al. [144] prepared piezoelectric and triboelectric composite smart cotton socks. The rubbing of fabric generated electrical signals during the movement of the human body. Due to its piezoelectric properties, the PZT was placed on the heel. When the device is worn, the pressure of the human body causes the PZT-based piezoelectric module to generate electrical signals.

### 4.2. Intelligent Prostheses and Human–Machine Interfaces

**Figure 11:**
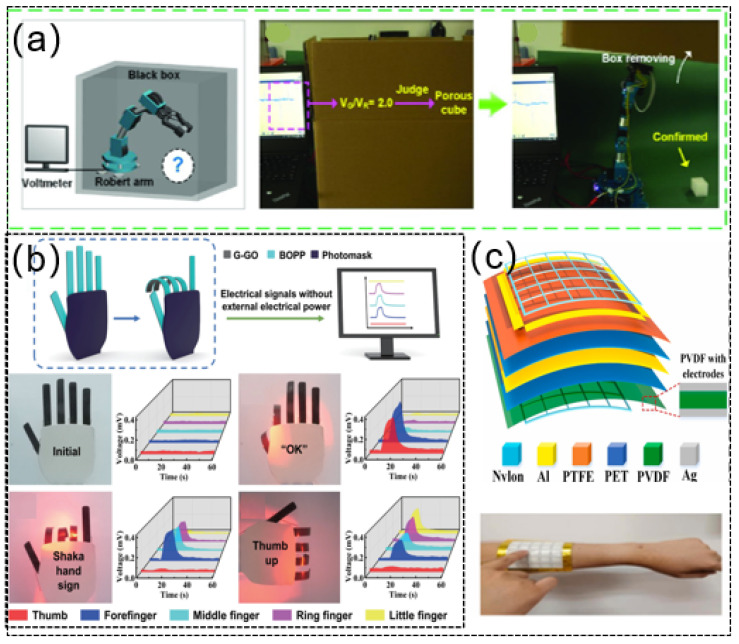
(**a**) Perception and learning function of intelligent prosthesis. Reprinted from Ref. [147]. (**b**) Bionic five-fingered hand. Reprinted with permission from Ref. [148]. Copyright 2021 Springer. (**c**) Schematic diagram of human–machine interface applied to arm. Reprinted with permission from Ref. [150]. Copyright 2021 Elsevier.

With the development of flexible sensors, they are becoming more and more important in intelligent prostheses, self-charging power cells for healthcare monitoring, and biological sensors [146]. Ma et al. [147] proposed self-powered soft tactile sensors using flexible magnetoelectric materials. The shape design of the flexible sensor was inspired by Merkel’s discs, allowing for good tactile perceptual functionality. This self-powered sensor is applied to the end of the manipulator, enabling the intelligent prosthesis to learn and distinguish between different objects after contact due to the flexible magnetoelectric materials used in the device. The equipment consists of two parts: the magnetic component mimicking Merkel’s disc at the top and the electrical control part at the bottom. In the process of grasping an object many times, its characteristic signals can be learned (Figure 11a). A self-powered actuator with an integrated sensing function plays an important role in the construction of an intelligent robot. Lin et al. [148] reported a self-powered actuator driven by light. A thermoelectric (TE) generator for collecting and converting environmental heat energy was designed using the thermoelectric effect. Due to the photoelectric effect, there is a temperature difference in the actuator, and a voltage signal is generated. The sensor consists of two parts. The first layer is the PTE generator, which is composed of graphene (G) and graphene oxide (GO). The second layer is a biaxially oriented polypropylene (BOPP) film with a large coefficient of thermal expansion (CTE). Infrared light is used to irradiate part of the actuator, resulting in a temperature rise in the irradiated part and a temperature difference across the whole actuator. The PTE effect spontaneously generates a self-supply voltage signal in the device. Due to the different expansion coefficients of the graphene composite layer and BOPP layer, the system changes, and the radiation part of the brake shows bending deformation (Figure 11b).

In the era of artificial intelligence, human–machine interfaces play a particularly irreplaceable role in the diverse interactions between man and machine [149]. Shi et al. [150] presented a simple self-powered interactive patch combining triboelectric and piezoelectric sensing mechanisms. It can not only detect a single-point contact of the finger but also detect continuous finger sliding through multiple point contacts separated along the contact trajectory. These results showed that the self-powered interactive patch based on triboelectric and piezoelectric sensing mechanism has high applicability and real-time practicability in various human–machine interfaces, such as energy acquisition, writing boards, automatic control, robots, virtual reality, augmented reality, and wearable electronic devices (Figure 11c). Dong et al. [151] proposed a self-powered wearable keyboard (SPWK) fabricated by integrating a large-area F-Teng sensor array. It can not only follow electrical signals but also recognize individual typing characteristics through a Haar wavelet. Considering its wearability, self-security, and power supply, the SPWK has practical application value in human–computer interaction equipment and personal user identification systems, where it can dynamically identify operating users.

### 4.3. Robotics

Sensors play a crucial role in robot control. In recent years, TENGs have been applied to robots. In the process of robot motion, it can provide feedback in terms of external force, speed, displacement, and other information to better control robot motion. This section mainly summarizes the application of self-powered flexible sensors in bionic robots, soft robots, and industrial robots.

**Figure 12:**
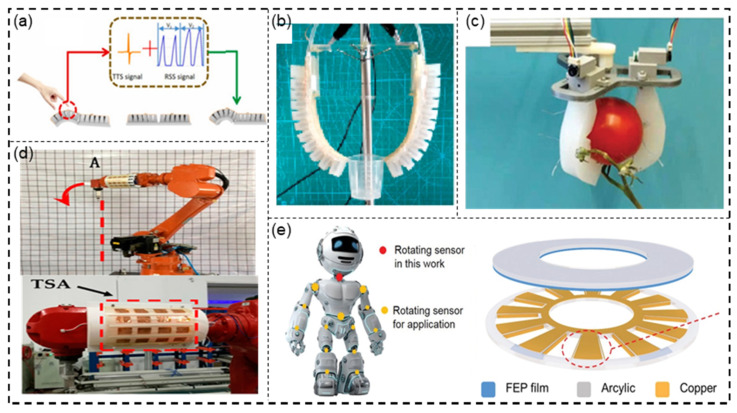
Robot applications. (**a**) Photograph of SCR. Reprinted with permission from Ref. [152]. Copyright 2021 Elsevier. (**b**) Photograph of PSA holding a small cup. Reprinted with permission from Ref. [153]. Copyright 2020 Elsevier. (**c**) Photograph of soft actuator picking tomato from its stem. Reprinted with permission from Ref. [154]. Copyright 2020 John Wiley and Sons. (**d**) Photograph of TSA system for an industrial robot. Reprinted from Ref. [155]. (**e**) Photograph of TVS for monitoring the rotary motion of a robot. Reprinted with permission from Ref. [156]. Copyright 2020 John Wiley and Sons.

Jin et al. [152] first applied a TENG to a soft caterpillar robot (SCR), which is similar to a bionic robot. There were four triboelectric tactile sensors (TTSs) and two resistive strain sensors (RSSs) attached to sense the surroundings and deformation, respectively. Due to its powerful sensing capabilities, it can be used in unknown tunnel environments or other unknown and complex environments, where its flexibility allows it to avoid unexpected attacks (Figure 12a).

Due to the flexibility of the TENG sensor, it has been applied to many soft actuators or grippers. Chen et al. [153] designed a soft pneumatic actuator (PSA), where the inner wall of the actuator and the electrode formed a TENG sensor, and the corresponding sensory information was outputted using the TENG’s self-powered sensing principle. For example, when grabbing cups, the size and shape of different cups can be determined by analyzing the feedback voltage value; the PSA can also be used to identify different gestures. The bending angle of PSA can be known, and the gestures can be recognized by analyzing the feedback voltage value (Figure 12b). Chen et al. [154] also designed a smart soft actuator that combines a cable with a TENG, which can grasp various objects. Unlike the PSA, it is driven by a tiny direct current (DC) motor. There are two types of TENGs used in the soft actuator: the TENG located inside the actuators that measure the bending angle and another TENG, located outside the actuator, used to detect contact pressure. Thus, the actuator functions similarly to the PSA, except that it can monitor the rough contours of the object being touched because the TENG of the actuator measures the contact force distributed over the entire surface of the actuator, whereas the PSA only has sensing capability at the tip (Figure 12c).

In terms of industrial robots, Liu et al. [155] demonstrated a large-scale, flexible, and self-powered tactile sensing array (TSA) for industrial robot skin. In a complex and unpredictable working environment, if unknown obstacles are encountered, the TSA can detect a sharp rise in voltage signal, and the pulse signal can convert the work program of the industrial manipulator into an interrupt program, which can be used for emergency avoidance and stopping (Figure 12d). Yin et al. [156] reported a triboelectric vector sensor (TVS) based on a DC triboelectric nanogenerator. It can measure motion parameters such as displacement, velocity, acceleration, and angular velocity. Therefore, it can be used in robot joints to detect changes in joint angles (Figure 12e).

### 4.4. Implantable Devices

Implantable devices can obtain energy through organisms, such as converting the mechanical energy of the heartbeat and intestinal peristalsis or the chemical energy of the glucose redox reaction into electrical energy, which can be applied to physiological sensors or to power cardiac pacemakers.

**Figure 13:**
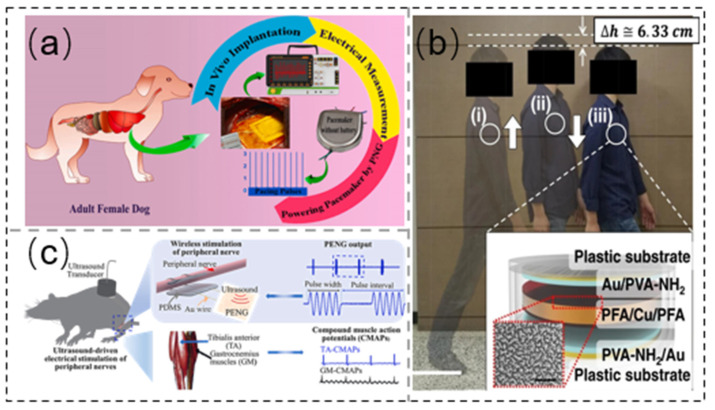
Implantable device applications. (**a**) Photograph of PNG attached to the LV wall. Reprinted with permission from Ref. [157]. Copyright 2021 Elsevier. (**b**) Photograph of a walker, along with schematic image of the I-TENG. Reprinted from Ref. [158]. (**c**) Photograph of nanogenerator implanted with a soft piezoelectric film to electrically stimulate peripheral nerves in rat. Reprinted with permission from Ref. [159]. Copyright 2021 Elsevier.

Most self-powered biomedical implants use piezoelectric energy collectors to collect electric energy from heart movement. Azimi et al. [157] demonstrated a piezoelectric polymer-based nanogenerator (PNG, a hybrid nanofiller composed of PVDF, ZnO, and rGO) that is biocompatible and flexible. The PNG converts the energy of the heart’s movement into electricity, and it is placed in the left ventricle to capture the amplitude of movement of the heart muscle. The PNG can extract 0.487 μJ of power from each heartbeat, which is higher than the pacing threshold of commercial pacemakers; hence, it can be used to power pacemakers (Figure 13a). In addition, Ryu et al. [158] reported a high-performance inertially driven triboelectric nanogenerator (I-TENG), approximately as large as a commercial coin battery. It converts the mechanical energy of body movement and gravity into electrical energy. I-TENG can generate 4.9 μW/cm^3^ of power. Therefore, a self-powered pacemaker can be formed by combining a pacemaker with I-TENG (Figure 13b). Chen et al. [159] studied a soft piezoelectric film nanogenerator for electrical stimulation of muscle nerves, which can be driven by a programmable ultrasonic pulse. In this paper, the sciatic nerve of rats was used as a model to achieve direct electrical stimulation via subcutaneous implantation of a piezoelectric membrane nanogenerator with a thickness of about 30 µm, driven by a remote ultrasonic pulse (Figure 13c).

In terms of implantable physiological sensors, Zhang et al. [160] reported a self-powered strain sensor based on electricity induced by the photoelectric thermoelectric (PTE) effect, which was composed of a stretchable graphene–ecoflex material. The strain sensitivity of the device increased with the increase in light intensity, exhibiting a sensor resolution of up to 0.5%, as well as a response time and recovery time of less than 0.6 s. Therefore, the device can monitor human joint movement and telescopic tension, with potential in implantable medical monitoring. Cheng et al. [161] reported a novel mechanical asymmetrical triboelectric nanogenerator for monitoring the intestinal state after glucose absorption. The lowest frequency that could be monitored in real time was 0.3 Hz. In this paper, ATNG was implanted into the abdominal cavity of rabbits to monitor duodenal peristalsis. After glucose absorption, duodenal peristalsis quickened and showed differences in terms of time and physiological state. The ATNG has great potential for the precise monitoring of weak motion in a variety of complex systems. Yang et al. [162] proposed a flexible self-powered implanted electronic skin (e-skin), which can be used to diagnose kidney diseases and analyze in situ body fluids (urea/uric acid content) in real time. The e-skin is made of piezoelectric ZnO nanowires, which exhibit flexibility, nontoxicity, and high energy conversion efficiency. In addition, its surface is modified by different enzymes (urease and uricase). Therefore, under the action of external forces, the output piezoelectric signal can represent the concentration information of urea/uric acid in body fluids as a function of the coupling process of the piezoelectric sensor and enzyme reaction.

### 4.5. AR/VR and Digital Twins

It is important in the future that robots can perceive and handle deformable objects. Thus, on machines such as grippers or gloves with TENG sensing, artificial intelligence can be realized through machine learning. It has excellent application prospects in unmanned supermarkets and man–machine communication.

**Figure 14:**
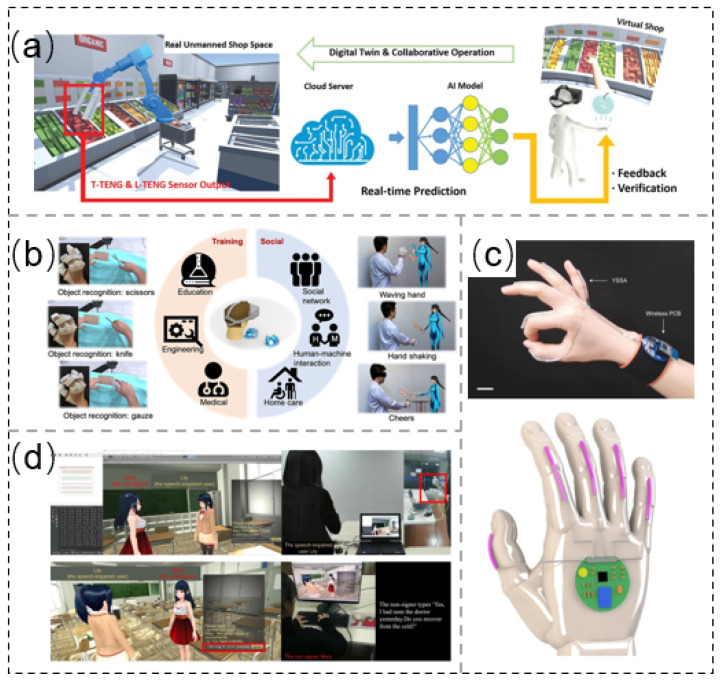
AI applications. (**a**) Photograph of digital-twin-based virtual shop system. Reprinted from Ref. [163]. (**b**) Photograph of various VR training programs and social activities using glove-based HMI. Reprinted from Ref. [164]. (**c**) Photograph of wearable sign-to-speech translation system and real-time display. Reprinted with permission from Ref. [165]. Copyright 2020 Springer. (**d**) Photograph of sign language recognition and communication system. Reprinted from Ref. [166].

Digital twins represent an emerging concept. Sun et al. [163] developed an intelligent soft robot manipulator. Combined with the Internet of things and artificial intelligence (AI), the manipulator can realize a virtual store based on digital twins and provide users with real-time feedback of product details. The manipulator is composed of a tactile TENG sensor (T-TENG), a length TENG sensor (L-TENG), and a polyvinylidene fluoride pyroelectric temperature sensor (PVDF). Twenty-eight objects of different shapes were captured several times, and data processing was carried out through machine learning (ML) with a precision of 97.143%. The temperature distribution of the target (resolution as low as 1 °C) could be obtained using the PVDF temperature sensor, so as to obtain more comprehensive information and realize automatic recognition of the captured target (Figure 14a).

Self-powered flexible sensors have also been developed for augmented reality/virtual reality (AR/VR) applications. Zhu et al. [164] proposed an intelligent glove that can be used for VR demonstration of surgical training programs and AR-based humanoid interaction. The smart glove uses a triboelectric-based finger bending sensor, a palm-sliding sensor, and a tactile sensor based on a piezoelectric mechanical stimulator. Similarly, machine learning was used to achieve target recognition with 96% accuracy. This device could benefit the medical, industrial, and educational sectors (Figure 14b).

In terms of AI, Zhou et al. [165] proposed a code language translation system consisting of self-powered TENG gloves and wireless transmission modules. With the help of machine learning, 660 gestures of American Sign Language (ASL) could be accurately translated into speech, with a recognition rate of 98.63% and recognition time of less than 1 s (Figure 14c).

However, an effective and practical approach for real-time sentence recognition of sign language is still lacking, which is more significant for the practical communication of signers and non-signers. Feng et al. [166] improved the performance of the sign language translation system through a non-segmentation and segmentation-assisted deep learning model, allowing the recognition of 50 words and 20 sentences. Through the segmentation model, new sentences generated by the reorganization of newly ordered words could be identified with an average accuracy of 86.67%. They translated sign language into text and audio presented in virtual space, allowing unrestricted communication between healthy and hearing/speech-impaired individuals (Figure 14d). Wang et al. [167] developed a silent speech strategy that does not require much sign language skills, allowing for all-day, natural interaction. It is attached to the facial skin; through the detection of face deformation, using the method of machine learning, it can recognize up to 110 words, basically covering daily vocabulary, with an average accuracy of 92.64%.

## 5. Summary and Outlook

We presented a brief overview of self-powered flexible sensors in terms of three aspects: material design, structural optimization, and frontier application. The emergence of PENGs and TENGs has been a major discovery in the field of self-powered flexible sensor. Recently, significant progress has been made with respect to self-powered flexible sensor in the fields of healthcare, robotics, industrial application, etc. These application fields also put forward higher requirements for self-powered flexible sensors, which are to be highly sensitive, accurate, stable, and mechanically flexible. Compared with conventional flexible sensors with an external battery, self-powered flexible sensors can effectively address the shortcomings of a battery with a limited lifetime. Moreover, TENGs and PENGs can solve the unpredictability of energy conversion in some special environments. Self-powered flexible sensors can significantly improve the performance of sensing to a certain extent. Therefore, as an effective conversion technology from mechanical energy to electrical energy and signals, PENGs and TENGs can be simultaneously used as sensing devices and energy sources. Self-powered flexible multifunctional sensors based on TENG and PENG have properties such as stretchability, self-healing, and degradability, and they have been applied in many fields, including wearable devices, electronic skin, intelligent prostheses, and human–machine interfaces. Furthermore, implantable devices have great potential in the medical and healthcare fields. Some researchers have achieved expected results in communication with a computer for construction of the mobile Internet of things, which has great value.

It is undeniable that huge progress has been made in self-powered flexible sensors, but there is a long way to go to translate existing research into practical value. Self-powered flexible sensors based on PENGs and TENGs with high output performance have a lot of room for improvement. Their higher output performance is conductive to the realization of wireless energy transmission. However, several reports about alternating current (AC)-based TENGs have only described AC–DC conversion by rectification. In the future, it is also urgent to improve TENG technology in terms of output performance and output modes. For example, an inverting TENG (I-TENG) can realize the AC mode through the coupling of triboelectrification and air breakdown (DC mode). Unlike AC-TENG, I-TENG delivers unique characteristics and performance parameters (width ratio and amplitude ratio of AC signals) that can be controlled. Benefiting from its novel output mode and outstanding output performance, the inverting TENG can be useful in self-powered controllers, intelligent systems, etc. Furthermore, several issues need to be studied and improved, such as sensitivity, stability, durability, and industrial production. In the context of artificial intelligence, how to effectively combine self-powered flexible sensors with human–machine interaction, intelligent robots, AR/VR, and other intelligent fields requires more research. TENGs and PENGs mainly convert mechanical energy into electrical energy. At present, there are also nanogenerators that convert wind energy, kinetic energy from seawater, and heat energy into electrical energy. However, the energy formed by these nanogenerators is not stable; therefore, it is of importance to investigate the stable conversion of other energy sources into electrical energy.

## Figures and Tables

**Table 1:** Material system and performance of self-powered stretchable flexible sensors with different shapes and structures [41,42,43,44,45,46,47,48,49,50,51,52,53,54].

Structure	Material	Max Strain	Stable Working State	Open-Circuit Voltage	Short-Circuit Current	Max Out Power	Self-Powered Mechanism
Fiber-shaped	PVDF/rGO/BT	≥100%	100%	1.3 V	/	/	PENG
	PTFE/LM	≥560%	/	490 V	/	/	TENG
	PVDF–HFP–TFE/LM	1170%	/	5.11 V	93 nA	/	TENG
	PVA/SEBS	≥1900%	/	/	10 nA	10.2 μW/m^2^	TENG
	Zn/SA/PAA	>10,000%	/	9.7 V	/	32 μW/m^2^	TENG
Flat-shaped	AL/PPy/Au	≥20%	20%	2.4 V	/	2450 μW/cm^2^	P-TENG
	AgNW/PEDOT/H-PDMS	≥50%	50%	100 V	/	327 mW/m^2^	TENG
	AgNWs/BaTO_3_/PDMS	≥60%	60%	105 V	/	102 mW/cm^2^	TENG
	AgNWS/Silicone rubber/Ni foam	≥100%	100%	12–15 V	60–80 nA	/	TENG
	PTFE/Elastuc rubber/Al	≥100%	120%	/	/	/	TENG
	PEO/WPU/PA/LTV silicon rubber	318%	/	197 V	17.3 μA	2.3 W/m^2^	TENG
	PDMS/OMMT-CTAB/AgNWS/ZnONWs	580%	/	160 V	14.2 μA	0.087 mW/cm^2^	TENG
	CB/TPU	646%	/	41 V	0.262 μA	/	TENG
	Gel–TA	1600%	/	1.12 V	/	/	PENG

**Table 2:** Comparisons of piezoelectric and triboelectric self-powered sensors made from different degradable materials [27,67,72,73,77,78,79,80].

Degradable Material Type	Voltage and Current	Power Density or Power	Application Scenario	Self-Powered Mechanism
Animal-Based	13 V, 0.4 μA	0.8 W/m^2^	LEDs, digital watch, touch perception, switch for Internet of things	Bio-TENG
	50 V, 3 μA	/	Control switches, electrochromic automotive rearview mirror	SF-TENG
	22 V, 0.12 μA	97 μW/cm^3^	Capacitor, LEDs	CPENG
	49 V, 1.9 μA	6600 μW/cm^3^	Capacitor, LEDs	PCPENG
Plant-Based	1.3 V, 10^−4^ mA/cm^2^	1.33 mW/m^2^	Pressure sensor systems	TENG
	15.5 V, 3.3 μA	11.8 μW	/	C/BT-5 PENG
	21.6 V, 10 nC, 16 μW	16 μW	Capacitor, Smart roof tile	D-TENG
Artificial material	40 V, 1 μA	/	Tissue repair	BD-TENG-PLGA
	26 V, 0.4 μA	/	Tissue repair	BD-TENG-PVA
	40 V, 1 μA	/	Tissue repair	BD-TENG-PCL
	28 V, 0.6 μA	/	Tissue repair	BD-TENG-PHB/V
	28 V, 220 nA	/	Tissue repair (wound healing treatment)	BD-iTENG
